# Interactions between leaf phenological type and functional traits drive variation in isoprene emissions in central Amazon forest trees

**DOI:** 10.3389/fpls.2024.1522606

**Published:** 2024-12-24

**Authors:** Michelle Robin, Christine Römermann, Ülo Niinemets, Jonathan Gershenzon, Jianbei Huang, Bruce W. Nelson, Tyeen C. Taylor, Vinícius Fernandes de Souza, Davieliton Pinho, Lucas Falcão, Caroline Lacerda, Sérgio Duvoisin Júnior, Axel Schmidt, Eliane Gomes Alves

**Affiliations:** ^1^ Biogeochemical Processes Department, Max Planck Institute for Biogeochemistry, Jena, Germany; ^2^ Institute for Ecology and Evolution, Friedrich-Schiller University, Jena, Germany; ^3^ German Centre for Integrative Biodiversity Research (iDiv), Halle-Jena-Leipzig, Germany; ^4^ Senckenberg Institute for Plant Form and Function (SIP), Jena, Germany; ^5^ Crop Science and Plant Biology Department, Estonian University of Life Sciences, Tartu, Estonia; ^6^ Department of Biochemistry, Max Planck Institute for Chemical Ecology, Jena, Germany; ^7^ Environmental Dynamics Department, National Institute of Amazonian Research, Manaus, Brazil; ^8^ Department of Civil and Environmental Engineering, University of Michigan, Ann Arbor, MI, United States; ^9^ Department of Tropical Forest Sciences, National Institute of Amazonian Research, Manaus, Brazil; ^10^ Department of Chemistry, University of Amazonas State, Manaus, Brazil

**Keywords:** terpenes, phenolics, leaf traits, Amazon trees, biogenic volatile organic compounds, BVOCs

## Abstract

The Amazon forest is the largest source of isoprene emissions, and the seasonal pattern of leaf-out phenology in this forest has been indicated as an important driver of seasonal variation in emissions. Still, it is unclear how emissions vary between different leaf phenological types in this forest. To evaluate the influence of leaf phenological type over isoprene emissions, we measured leaf-level isoprene emission capacity and leaf functional traits for 175 trees from 124 species of angiosperms distributed among brevideciduous and evergreen trees in a central Amazon forest. Evergreen isoprene emitters were less likely to store monoterpenes and had tougher and less photosynthetically active leaves with higher carbon-to-nitrogen ratios compared to non-emitters. Isoprene emission rates in brevideciduous trees were higher with a higher diversity of stored sesquiterpenes and total phenolics content. Our results suggest that the way isoprene emissions relate to growth and defense traits in central Amazon trees might be influenced by leaf phenological type, and that isoprene may participate in co-regulating a chemical-mechanical defense trade-off between brevideciduous and evergreen trees. Such knowledge can be used to improve emission estimates based on leaf phenological type since, as a highly-emitted biogenic volatile organic compound (BVOC), isoprene affects atmospheric processes with implications for the Earth’s radiative balance.

## 1 Introduction

Volatile isoprenoids (VIs; isoprene, monoterpenes, and sesquiterpenes) emitted by plant leaves constitute the largest share of global Biogenic Volatile Organic Compound (BVOC) emissions (Guenther et al., 2012), being involved in a wide range of processes from plant cell regulation to forest-atmosphere interaction dynamics. On the individual scale, isoprene (C_5_H_8_) has been assigned numerous roles in plant growth and defense responses. Its association with increased thermotolerance (Singsaas et al., 1997; Pollastri et al., 2014, 2019) has led to different mechanistic hypotheses, from improved thylakoid membrane stability (Velikova et al., 2011; Harvey et al., 2015), to direct antioxidant activity (Velikova, 2008) and serving as a sink of excessive reducing power (Morfopoulos et al., 2013, 2014; Rodrigues et al., 2020). Currently, multi-omic studies suggest more complex associations between the presence of isoprene emissions and multiple signaling networks, linking it to changes in transcription factors involved in plant growth and in the production of defense and stress tolerance compounds (Behnke et al., 2010; Harvey and Sharkey, 2016; Lantz et al., 2019; Zuo et al., 2019; Frank et al., 2021; Monson et al., 2021; Dani et al., 2022; Weraduwage et al., 2023; Srikanth et al., 2024). On the scale of plant populations, species, and communities, monoterpenes (C_10_H_16_) and sesquiterpenes (C_15_H_24_) - and recently isoprene - are suggested to have diverse chemical signaling roles in direct and indirect defense against herbivory, plant-plant communication, and attraction of pollinators (Pichersky and Gershenzon, 2002; Gershenzon and Dudareva, 2007; Laothawornkitkul et al., 2008; Fineschi and Loreto, 2012; Xiao et al., 2012; Monson et al., 2021). On regional and global atmospheric scales, VI emissions have an impact on the oxidative capacity of the atmosphere as these compounds are rapidly oxidized and decomposed in the presence of ozone (O_3_), hydroxyl radical (OH), and nitrogen oxides (NO_x_), and can influence light scattering and precipitation through the formation and growth of secondary organic aerosols and cloud condensation nuclei (Griffin et al., 1999b, 1999a; Kuhn et al., 2007; Lelieveld et al., 2008; Pöschl et al., 2010; Kulmala et al., 2013; Pfannerstill et al., 2018; Yáñez-Serrano et al., 2020).

Isoprene and monoterpenes are produced in the chloroplast of leaves through the methyl-erythritol 4-phosphate (MEP) pathway (Zhao et al., 2013), while sesquiterpenes are produced in the cytosolic mevalonic acid (MVA) pathway (Vranová et al., 2013). About 90% of isoprene production originates from recently assimilated photosynthetic carbon under non-stressful conditions (Delwiche and Sharkey, 1993; Affek and Yakir, 2003; Loreto et al., 2004; Sharkey and Monson, 2017), although there can be alternative carbon sources under stress (Kreuzwieser et al., 2002; Funk et al., 2004; Schnitzler et al., 2004; Jardine et al., 2014; de Souza et al., 2018). Similar to isoprene, light-dependent constitutively-emitted monoterpenes are produced and emitted from recently assimilated carbon in some plant species, although much less frequently than isoprene (Loreto et al., 1996; Jardine et al., 2017). More frequently, monoterpenes and sesquiterpenes form storage pools in the cell wall or specialized storage structures (e.g., resin ducts, oil glands, glandular trichomes) and are released slowly under constitutive conditions or emitted rapidly upon breakage of these structures (e.g., under herbivore feeding) (Arneth and Niinemets, 2010; Niinemets et al., 2013; Rasulov et al., 2019; Nagalingam et al., 2023).

The generally observed light and temperature dependence of VI emissions makes tropical forests the largest source of global fluxes, accounting for around 80% of global BVOC emissions (Guenther et al., 2012). In addition, recent studies have reported that c. 76% of tropical forest tree species are isoprene emitters (Mu et al., 2022). Considering its high plant biomass and species diversity (Fauset et al., 2015; Cardoso et al., 2017), the Amazon forest can be the greatest and most diverse - in terms of compound diversity - source of VI emissions (Yáñez-Serrano et al., 2020; Gomes Alves et al., 2023). Measuring leaf-level VI emission at remote and often inaccessible locations of the Amazon forest is logistically challenging but fundamental to identify the factors that determine global isoprene emissions, and to improve emission predictions considering global changes in temperature and precipitation. Besides light and temperature, another important driver of isoprene emissions is leaf age and possibly different leaf phenological types (Dani et al., 2014), and seasonal variation in emissions in the central Amazon forest was shown to be determined by leaf age and leaf flushing events (Alves et al., 2014, 2016, 2018; Gomes Alves et al., 2023). More specifically, this variation has been attributed to age-driven changes in leaf physiology and tree crown architecture: concerning leaf physiology, the activity of isoprene synthase is lower or even absent in young leaves, peaking in mature leaves and decreasing with leaf senescence (Schnitzler et al., 1997); concerning tree crown architecture, older leaves of evergreen trees may experience lower amounts of intercepted light because of shading caused by the flushing of new leaves, leading to lower emission rates (Niinemets et al., 2004, 2010). Other studies have proposed that isoprene emissions are probably replaced by emissions of stored terpenes in evergreen plants as a way to better handle recurrent and extended periods of stress or that, compared to evergreen species, deciduous plants would be higher isoprene emitters due to associations between emissions, resource-acquisition strategies, and shorter leaf lifespan (Harrison et al., 2013; Dani et al., 2014, 2022), but these studies tend to be biased by temperate forest tree species due to the larger data availability for these forests.

Different from temperate forests, where leaf flushing is mostly determined by temperature seasonality (Perry, 1971), leaf flushing in Amazon forests is determined by precipitation seasonality, with massive flushing crowns occurring during the driest months (Lopes et al., 2016; Wu et al., 2016; Aleixo et al., 2019). Also, in temperate forests, deciduous trees lose all of their foliage and remain bare for several months, while in central Amazon forests, deciduousness is more subtle: brevideciduous trees may not lose all their foliage at the same time, and tree crowns become fully deciduous for shorter periods, of up to one month before flushing a new cohort of leaves (Lopes et al., 2016; Gonçalves et al., 2020). Previous leaf-out phenology studies in central Amazon forests have shown the co-occurrence of brevideciduous and evergreen trees, with a prevalence of evergreen over brevideciduous trees (Condit et al., 2000; Aleixo et al., 2019). Brevideciduous trees lost part or all of their foliage and flushed new leaves concentrated in the drier months of the year, whereas evergreen trees were divided into trees that had detectable but irregular flushing events and massively flushed new leaves - predominantly in the drier months of the year - and trees that did not show visually detectable flushing events and lost and produced leaves more gradually throughout the year (Gonçalves et al., 2020; Mesquita Pinho, 2021; Botía et al., 2022). Recently, Gomes Alves et al. (2023), examined the isoprene emission trait for 194 PhenoCam-monitored trees in a central Amazon forest and observed similar fractions of potential isoprene emitters in all leaf phenological types, yet leaf-level measurements and variations in isoprene emission rates between different leaf phenological types in this forest have not been done or evaluated.

Considering the importance of climate seasonality, leaf age, and possibly leaf phenological type over isoprene emissions in Amazon forests, our study seeks to evaluate whether leaf phenological type and leaf functional traits drive variation in the presence and magnitude of isoprene emission capacity (*E*_c_; emission measured at standard conditions: light of 1000 µmol m^-2^ s^-1^ photosynthetically active radiation and leaf temperature of 30°C) and terpene storage in central Amazon trees. We measured leaf-level isoprene *E*_c_ and leaf functional - physiological, morphological, and chemical - traits for 175 trees from 124 species of angiosperms distributed among brevideciduous and evergreen trees in a central Amazon forest. Because isoprene is lighter in terms of carbon atoms per molecule (C5), non-storable in leaves, and has been associated with resource-acquisition strategies and shorter leaf longevity, we hypothesized that a higher presence and/or magnitude of isoprene emissions would be associated with a brevideciduous behavior (i.e., annual leaf turnover); at the same time, because monoterpenes and sesquiterpenes are heavier (C10 and C15), can be stored inside the leaves, serve as herbivore deterrents, and have been associated with resource-conservation strategies and higher leaf longevity, we hypothesized that a higher presence and/or magnitude of their storage would be associated with evergreen trees (Wright et al., 2004; Harrison et al., 2013; Dani et al., 2014).

## 2 Materials and methods

### 2.1 Study site

We performed measurements in an upland forest (locally called *terra firme*) permanent plot at the Amazon Tall Tower Observatory (ATTO) site in central Amazonia. The ATTO site is located about 150 km northeast of Manaus in the Uatumã Sustainable Development Reserve (02° 08.9’ S, 59° 00.2’ W, 130 m a.s.l.). The site is situated in a humid tropical climate zone, with a mean annual temperature of 26.7°C and precipitation of 2376 mm and characterized by a pronounced wet season from December to May and a dry season from July to October, with a transitory moderately wet period in between the seasons (Botía et al., 2022). Vegetation in the *terra firme* plot is dense (leaf area index of 5.3 m^2^ m^-2^), mature, and non-flooded, with a mean canopy height of 35 m (Gomes Alves et al., 2023). The soil is a highly weathered and well-drained ferralsol (Chauvel et al., 1987). More details on the experimental site are provided by Andreae et al. (2015).

### 2.2 Leaf phenological type

Located inside the *terra firme* plot is an 80 m high tower (INSTANT, 02°08.7520′ S, 58°59.9920′ W) with a StarDot RGB camera (model NetCam XL 3MP) installed on top of it at 81 m height facing west. For more details on the camera setup, radiometric calibration, and detection of phenological stages see Lopes et al. (2016). The camera (PhenoCam) monitored upper-crown surfaces of 194 liana-free trees from July 2013 to November 2018 generating an image-derived leaf longevity dataset (i.e., PhenoCam dataset) that allowed the classification of trees into three categories of leaf phenological type which were defined as follows: i) brevideciduous (BD) - trees that lost all of their foliage/part of their foliage and flushed new leaves concentrated in the drier months of the year; ii) evergreen (EV) - trees that showed detectable flushing events and massively flushed new leaves, predominantly in the drier months of the year; and iii) no flushing detected (NF) - evergreen trees that possibly added and lost leaves throughout the years and did not show detectable flushing crown events during the monitoring period (Botía et al., 2022). Leaf phenological type classifications agree with satellite vegetation indices retrieved from MODIS-MAIAC (Multi-Angle Implementation of Atmospheric Correction) for this region (Gonçalves et al., 2020) and branch-level monitoring of leaf age distributions for trees from this plot (Gomes Alves et al., 2023). At the PhenoCam view, the BD group contained 49 trees from 45 species, the EV group 83 trees from 60 species, and the NF 62 trees from 53 species. Only 36 species in the dataset had replicate trees available, and trees of the same species showed different leaf phenological types. Such intra-specific variability in leaf phenological type has been observed in another tropical forest (Park et al., 2019). Moreover, leaf phenological types are subject to phenotypic plasticity, and studies have observed that “random” events such as herbivore attacks, pathogens, and environmental changes caused by extreme events can alter leaf-out phenology patterns in some trees (Borchert, 1999; Cleland et al., 2007; Gonçalves et al., 2020).

### 2.3 Branch collection

Of the 194 trees in the PhenoCam dataset, we were able to sample branches from 175 trees from 124 species of angiosperms. All of the trees occupied the upper canopy layer of the plot and were the most representative in terms of canopy dominance. For all trees, we measured diameter at breast height (DBH, diameter at 1.3 m height), leaf-level isoprene *E*_c_, net photosynthesis rate (*A*_n_), leaf morphological traits (leaf dry matter content, LDMC; leaf mass per area, LMA; leaf thickness, LT; leaf toughness or Force to Punch, FtP) and collected leaves for leaf stable C isotope and elemental analyses, terpene (mono- and sesquiterpene) storage analysis and total phenolics content analysis. We sampled the trees and performed measurements between October 15 - November 9, 2022. This period corresponds to the transition between dry and wet seasons, when tree canopies are mostly composed of mature leaves (Alves et al., 2018; Gonçalves et al., 2020), and variation in leaf age is expected to be low.

Given the logistical challenges of studying tall tropical trees, often exceeding 20 meters in height, leaf measurements were obtained from cut branches immediately placed in water. This method provides a practical solution for conducting gas exchange and isoprene emission measurements, enabling the capture of key ecological processes without compromising leaf viability (Llusia et al., 2014; Albert et al., 2018; Jardine et al., 2020; Taylor et al., 2021; Gomes Alves et al., 2022). Branches with diameters of at least 2 cm were collected from sun-exposed areas of the canopy to avoid shade-adapted leaves. Senescent, immature, or visibly damaged leaves were excluded, ensuring that only physiologically active leaves were analyzed. After collection, branches were immediately re-cut underwater to prevent embolism formation in open vessels, stored in water bottles for transport, and re-cut once more under water at the field camp to restore xylem flow before isoprene *E*_c_ and gas exchange measurements (section 2.5).

### 2.4 Leaf samples for isoprene emission capacity and functional trait measurements

We selected one visibly mature and healthy leaf of the branch to measure leaf-level isoprene *E*_c_ and *A*_n_, then removed the branch from the water, wrapped the lower end of the stem in moist absorbent paper, and placed it in a closed plastic bag for further leaf morphological trait measurements. We selected between 10-20 leaves (fewer larger leaves and more smaller leaves were collected) that were immediately frozen in liquid nitrogen and further taken to Manaus for terpene storage analysis and selected another set of 10-20 leaves that were dried in an oven at 60°C for 72 h, ground and weighed for leaf stable C isotope and elemental analyses and total phenolics content analysis at the Max Planck Institutes for Biogeochemistry (MPI-BGC) and Chemical Ecology (MPI-CE). Finally, we selected four leaves (including the one used to measure isoprene *E*_c_) to measure LDMC, LMA, and LT, and another four leaves to measure FtP. For compound leaves, we considered a leaflet as an equivalent of a simple leaf for all leaf measurements described below. Detailed descriptions of leaf morphological trait measurements, terpene storage analysis, stable C isotope and elemental analyses, and total phenolics content analysis are presented in the Supplementary Material (**Supplementary Methods S1–S4**).

### 2.5 Isoprene emission capacity and gas exchange measurements

We measured leaf-level isoprene *E*_c_ using a combined LI-6800 portable gas exchange (LiCor Inc., USA) and proton-transfer-reaction quadrupole mass spectrometer (PTR-QMS, IONICON Analytik, Innsbruck, Austria) system, which allows real-time measurements of isoprene emissions under defined environmental conditions of the LI-6800 leaf chamber. We installed a hydrocarbon filter (Restek Pure Chromatography, Restek Corporations, USA) at the air inlet of the LI-6800 to remove isoprene from incoming ambient air. All tubing in contact with the sampling air was PTFE and does not exchange isoprene. At the beginning of each day and before each measurement, we obtained a chamber blank sample from the empty leaf chamber. We separately enclosed the leaf (for compound leaves we considered a leaflet as the equivalent of a simple leaf lamina) in the leaf chamber under standard conditions: photosynthetic photon flux density (PPFD) of 1000 μmol m^-2^ s^-1^, leaf temperature of 30°C, flow rate of air going into the leaf chamber of 400 μmol s^-1^, CO_2_ and H_2_O concentrations of 420 μmol mol^-1^ and 21 mmol mol^-1^ and relative humidity of ~60%. The stability criterion for measurements was defined as one standard deviation of the mean *A*_n_, and we visually monitored *A*_n_ until the value reached a plateau, beginning measurements when the instrument had reached the defined stability criterion. Leaves displaying no signs of photosynthetic activity were excluded from analyses. *A*_n_ was transformed to photosynthesis per leaf dry mass (*A*_mass_) and expressed in units of μg C g^-1^ h^-1^.

The air exiting the LI-6800 leaf chamber was redirected to the PTR-QMS, which operated in standard conditions with a drift tube voltage of 600 V, drift tube pressure of 2.2 mbar, and E/N 120 Td. Measurements were performed for 10 minutes, and during each PTR-QMS measurement cycle the following mass-to-charge ratios (m/z) were monitored: 21 (H_3_^18^O_+_), 32 (O_2_^+^), and 37 (H_2_O-H_3_O^+^) with a dwell time of 500 ms each; 41 (isoprene fragment), 69 (isoprene) with a dwell time of 1 s each. Humidity-dependent calibrations (using water-bubbled nitrogen to dilute standard gas, simulating ambient relative humidity) were performed with a certified standard gas provided by Apel-Riemer Environmental, Inc. (**Supplementary Table S1**), at the beginning and end of the measurement campaign. The mixing ratios of isoprene were calculated from the calibration curves (R^2^ = 0.99). The detection limit of the PTR-QMS was calculated as three times the standard deviation of isoprene (ppb) detected in the water-bubbled nitrogen background of the calibration curves and was equal to 0.93 ppb. Cross-validation for isoprene data obtained by *in situ* PTR-QMS measurements and by adsorbent cartridges analyzed via GC-FID was performed in a previous study showing a coefficient of determination (*r* ^2^) of 0.88 (Yáñez-Serrano et al., 2015). Once mixing ratios of isoprene (ppb) from the samples were obtained, isoprene emission capacity per area *E*_c,A_ was determined using the equation (*E*_c,A_ = *Rppb* × Q/S), where *E*_c,A_ (nmol m^-2^ s^-1^) is the leaf flux of isoprene emission; *Rppb* (nmol mol^-1^) is isoprene concentration of the outgoing air; Q is the flow rate of air into the leaf chamber (400 x 10^-6^ mol s^-1^); S is the area of leaf within the chamber (0.0002 m² or 0.0006 m²). Values of isoprene *E*_c,A_ were transformed to units of isoprene emission capacity per dry mass (*E*_c,M_, µg C g^-1^ h^-1^).

### 2.6 Statistical analyses

Because the number of replicates per species available in our sampling plot prevented characterizing species-level variation, we focused on individual-level analyses and controlled potential species-level effects by performing mixed-effects models with species as random factor. To evaluate if the presence of isoprene emissions or terpene storage changed between leaf phenological types, we performed chi-squared (χ^2^) analysis to compare observed and expected proportions of detected isoprene *E*_c,A_, and mono-/sesquiterpene storage in the full dataset and to compare observed and expected proportions of detected isoprene *E*_c,A_ and mono-/sesquiterpene storage in each leaf phenological type.

To evaluate if the magnitudes of isoprene emissions and terpene storage changed between leaf phenological types, we performed mixed-effects pair-wise comparisons of the magnitude of isoprene *E*_c,A_ and relative abundances of stored mono-/sesquiterpenes between leaf phenological types. To evaluate whether the interactions between leaf phenological types and functional traits influenced the presence of isoprene emissions or the variation in isoprene emission rates, we performed univariate mixed effects linear regression models (UMELMs) of detected isoprene *E*_c,M_/magnitude of isoprene *E*_c,M_ ~ functional trait * leaf phenological type + (1| Species). Given that some traits had missing data (NA), UMELMs were performed with a reduced sample size of *n* = 154 for detected isoprene *E*_c,M_ and *n* = 81 for the magnitude of isoprene *E*_c,M_ (only trees with detected isoprene *E*_c,M_). We performed univariate models instead of a single multiple model containing all functional traits measured because our number of observations did not allow for the inclusion of all these variables and their interactions in a single multiple model (Harrell, 2001; Burnham and Anderson, 2002; Babyak, 2004).

UMELMs were performed using the lmer function of the LME4 R package (Bates et al., 2015). The p-values of mixed effects pairwise comparisons and UMELMs were obtained with the EMMEANS package (Lenth, 2024). Distributions of detected isoprene *E*_c,A_, and mono-/sesquiterpene storage between leaf phenological types and results of mixed effects models are presented as plots from the GGPLOT2 package (Wickham, 2016). All statistical analyses were performed using R version 4.3.2 through the platform RStudio 2023.9.1.494 (R core team, 2023).

## 3 Results

**Figure 1:**
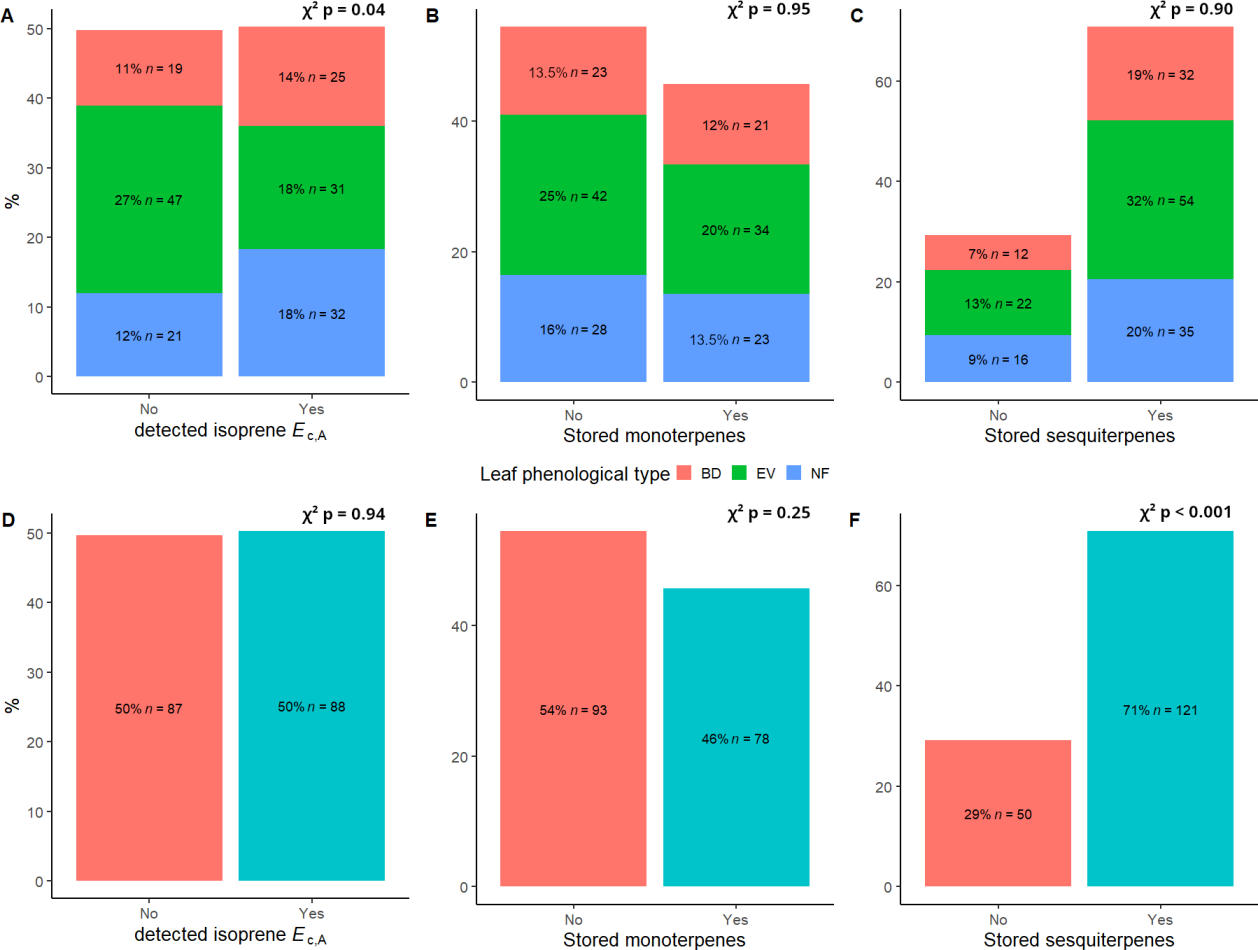
Distribution of **(A)** detected isoprene *E*_c,A_, **(B)** stored monoterpenes, and **(C)** stored sesquiterpenes between leaf phenological types and observed proportions of **(D)** detected isoprene *E*_c_, **(E)** stored monoterpenes and **(F)** stored sesquiterpenes for 175 trees from 124 species of angiosperms in a central Amazon forest. BD, brevideciduous, trees that lost all their foliage/part of their foliage and flushed new leaves concentrated in the drier months of the year; EV, evergreen, trees that showed detectable flushing events and massively flushed new leaves, predominantly in the drier months of the year; NF, no flushing detected, evergreen trees that possibly added and lost leaves throughout the year and did not show detectable flushing crown events during the monitoring period. Chi-squared (χ^2^) p-values in a-c correspond to comparisons between observed and expected proportions of emission/storage in each leaf phenological type (heat map of residuals for panel **(A)** is presented in **Supplementary Figure S1**), and χ^2^ p-values in d-f correspond to comparisons between observed and expected proportions of emission/storage in the full dataset.

**Figure 2:**
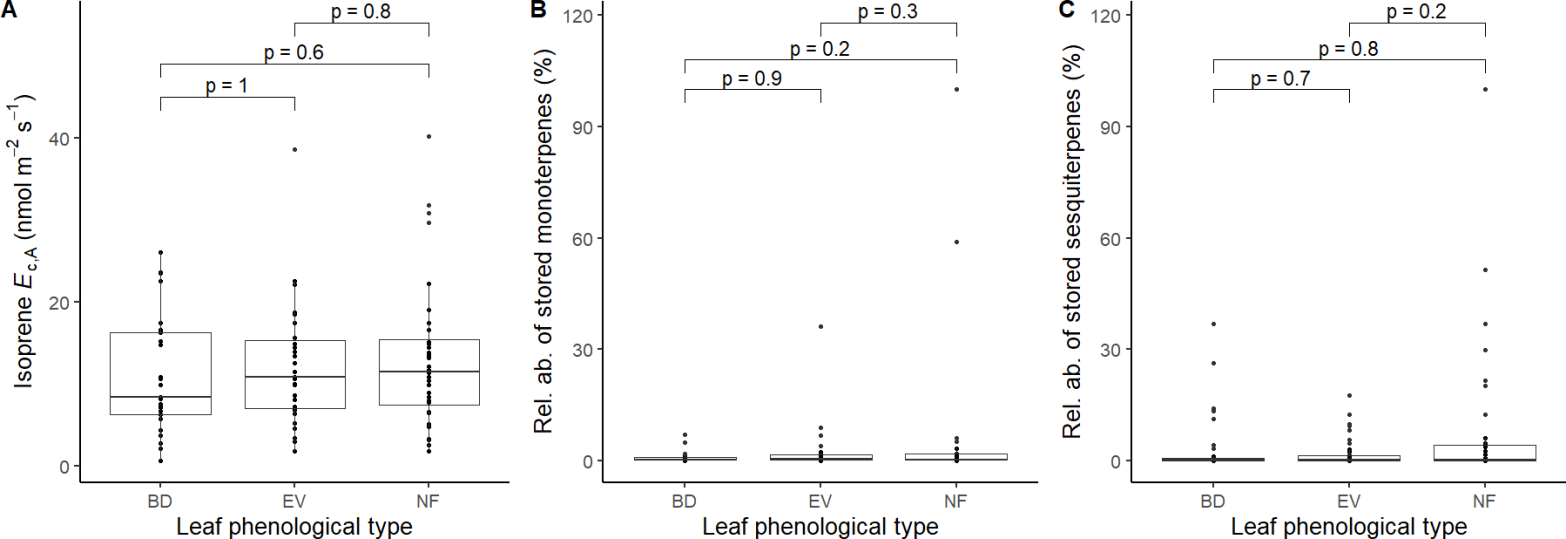
Comparisons of the magnitude of **(A)** detected isoprene *E*_c,A_ (nmol m^-2^ s^-1^, *n* = 88), **(B)** relative abundances of stored monoterpenes (%, *n* = 78), and **(C)** stored sesquiterpenes (%, *n* = 121) between leaf phenological types. BD, brevideciduous, trees that lost all their foliage/part of their foliage and flushed new leaves concentrated in the drier months of the year; EV, evergreen, trees that showed detectable flushing events and massively flushed new leaves, predominantly in the drier months of the year; NF, no flushing detected, evergreen trees that possibly added and lost leaves throughout the year and did not show detectable flushing crown events during the monitoring period. Pairwise comparisons are mixed effect models that include tree species as a random effect.

Values of mean, standard deviation, and ranges of values for all variables used in this study are presented in **Table 1**. We found a total of 14 different stored monoterpenes and 25 stored sesquiterpenes (**Table 2**). The distribution of detected isoprene *E*_c,A_ between leaf phenological types showed that there was a significantly higher occurrence of isoprene non-emitters among evergreen (EV) trees (chi-squared test (χ^2^), p = 0.04; **Figure 1A**). There were no significant differences in percentages of detected terpene storage comparing leaf phenological types (**Figures 1B, C**). We detected isoprene *E*_c,A_ in 88 trees (50%) (**Figure 1D**), monoterpene storage in 78 trees (46%) (**Figure 1E**), and sesquiterpene storage in 121 trees (71%) (**Figure 1F**), and there was a much higher number of sesquiterpene-storing trees than expected by chance (p < 0.001, **Figure 1F**). There were no significant differences in isoprene emission rates and relative abundances of stored terpenes between leaf phenological types (**Figure 2**).

**Table 1:** Units and values of mean, standard deviation (SD), and range of values in the dataset for isoprene emission capacity per area (*E*_c,A_) and per leaf dry mass (*E*_c,M_) and leaf functional traits measured for 175 trees from 124 species of angiosperms in a central Amazon forest.

Variable	Unit	Mean	SD	Range of values
Isoprene emission capacity per area (*E*_c,A_)	nmol m^-2^ s^-1^	6.2	8.4	0 - 40.1
Isoprene emission capacity per dry mass (*E*_c,M_)	µg C g^-1^ h^-1^	1.8	2.4	0 - 11.5
Leaf dry mass per area (LMA)	g cm^-2^	0.08	0.04	0.03 - 0.4
Leaf dry matter content (LDMC)	mg g^-1^	469.9	76.1	216.1 - 698.7
Leaf thickness (LT)	mm	0.2	0.07	0.1 - 0.6
Force to Punch (FtP)	N mm^-1^	0.3	0.1	0.04 - 0.5
Carbon-to-nitrogen ratio (CN)		27.7	8.0	8.6 - 52.8
Phosphorus concentration (*P*_mass_)	mg g^-1^	0.6	0.3	0.2 - 2.1
Foliar δ^13^C	‰	-30.9	1.5	-34.4 - -27.2
Net photosynthesis per area (*A*_n_)	μmol m^-2^ s^-1^	3.7	3.5	0.004 - 15.0
Photosynthesis per mass (*A*_mass_)	µg C g^-1^ h^-1^	223.2	222.0	0.303 - 1098.1
Relative abundance of stored monoterpenes	%	1.6	9.3	0 - 100
Relative abundance of stored sesquiterpenes	%	2.9	10.3	0 - 100
Total phenolics	%	8.4	15.0	0 - 100
Stored monoterpene diversity	*n* of compounds	1.5	2.4	0 - 13
Stored sesquiterpene diversity	*n* of compounds	3.1	3.6	0 - 15
Presence of stored monoterpenes		0.46	0.5	0 or 1
Presence of stored sesquiterpenes		0.71	0.5	0 or 1

Relative abundances of stored monoterpenes and sesquiterpenes are calculated as the sum of peak areas of stored monoterpenes (sum of stored monoterpenes) and stored sesquiterpenes (sum of stored sesquiterpenes) found in a given sample, normalized by the largest sum observed in the dataset for each group of compounds. Stored monoterpene and sesquiterpene diversity refer to the number of different mono- and sesquiterpene compounds found in each sample.

**Table 2:** List of detected stored monoterpenes and sesquiterpenes and number (*n*) of trees in which each compound was detected.

Monoterpenes		Sesquiterpenes	
Compound	*n* of trees	Compound	*n* of trees
Limonene	56	Caryophyllene	97
Linalool	39	Copaene	94
p-Cymene	35	α-Calacorene	31
α-Terpineol	33	Alloaromadendrene	31
α-Pinene	17	α-Cubebene	27
γ-Terpinene	16	α-Muurolene	24
β-Ocimene	13	*cis*-α-Bergamotene	23
Terpinen-4-ol	10	Globulol	22
Camphene	7	γ-Muurolene	18
Eucalyptol	7	Ylangene	17
α-Phellandrene	5	Aromandendrene	16
*p*-Menthatriene	5	Selina-3-7-11-diene	13
β-Myrcene	4	α-Guaiene	13
endo-Borneol	4	α-Maaliene	12
		τ-Muurolol	11
		Guaiol	11
		β-Bourbonene	10
		τ-Cadinol	8
		Humulene	8
		Isoledene	8
		γ-Elemene	7
		*trans*-Calamenene	7
		*cis*-Muurola-4-15-5-diene	6
		Neointermedeol	5
		β-Bisabolene	4

Results of UMELMs of detected isoprene *E*_c,M_ and functional traits (**Table 3**) showed that the interactions between leaf phenological type and Force to Punch (FtP), photosynthesis per mass (*A*_mass_), presence of stored monoterpenes, and carbon-to-nitrogen (CN) ratio were significantly related to the presence or absence of isoprene emissions (**Supplementary Table S2**). Meanwhile, results of UMELMs of the magnitude of isoprene *E*_c,M_, and functional traits (**Table 3**) showed that FtP, CN, and *A*_mass_ alone, and the interactions between leaf phenological type and the diversity of stored sesquiterpenes and total phenolics content, were significantly related to variations in isoprene emission rates (**Supplementary Table S2**).

**Table 3:** Statistical parameters of univariate mixed effects linear regression models (UMELMs) of detected isoprene *E*_c,M_ and magnitude of isoprene *E*_c,M_.

Dependent variable	Independent variables	df1	df2	F ratio	*p* trait	*p* Pheno.type	*p* interaction
Detected isoprene *E*_c,M_	FtP * Pheno.type	2	148	4.69	0.15	0.05	**0.01**
	*A*_mass_ * Pheno.type	2	148	4.62	0.1	0.06	**0.01**
	Presence of stored monoterpenes * Pheno.type	2	141	4.13	0.4	0.07	**0.02**
	CN * Pheno.type	2	146	3.05	0.9	0.05	**0.04**
	LT * Pheno.type	2	130	2.41	0.6	0.06	0.09
	Presence of stored sesquiterpenes * Pheno.type	2	148	2.32	0.4	0.2	0.1
	LDMC * Pheno.type	2	134	1.85	0.4	0.09	0.2
	Diversity of stored monoterpenes * Pheno.type	2	148	1.50	0.5	0.07	0.2
	LMA * Pheno.type	2	147	1.48	0.3	0.06	0.2
	Relative abundance of stored sesquiterpenes * Pheno.type	2	127	1.21	0.1	0.2	0.3
	Diversity of stored sesquiterpenes * Pheno.type	2	148	0.98	0.2	0.06	0.4
	Total phenolics * Pheno.type	2	140	0.57	0.8	0.07	0.6
	*P*_mass_ * Pheno.type	2	148	0.45	0.6	0.06	0.6
	Relative abundance of stored monoterpenes * Pheno.type	2	138	0.26	0.5	0.2	0.8
	δ^13^C* Pheno.type	2	137	0.10	0.2	0.08	0.9
Magnitude of isoprene *E*_c,M_	Diversity of stored sesquiterpenes * Pheno.type	2	60	4.82	0.9	0.7	**0.01**
	Total phenolics * Pheno.type	2	49	3.23	0.6	0.8	**0.05**
	LMA * Pheno.type	2	22	3.07	0.006	0.65	0.07
	Presence of stored sesquiterpenes * Pheno.type	2	50	2.35	1.0	0.3	0.1
	Relative abundance of stored sesquiterpenes * Pheno.type	2	54	1.35	0.2	0.9	0.3
	δ^13^C * Pheno.type	2	59	1.26	0.4	0.7	0.3
	Diversity of stored monoterpenes * Pheno.type	2	70	1.24	0.8	0.8	0.3
	FtP * Pheno.type	2	74	1.10	**0.01**	0.5	0.3
	CN * Pheno.type	2	68	0.88	**0.001**	0.9	0.4
	Relative abundance of stored monoterpenes * Pheno.type	2	66	0.70	0.8	0.9	0.5
	LT * Pheno.type	2	56	0.64	0.1	0.7	0.5
	LDMC * Pheno.type	2	60	0.48	0.07	0.7	0.6
	*A*_mass_ * Pheno.type	2	49	0.18	**0.0002**	0.6	0.8
	Presence of stored monoterpenes * Pheno.type	2	62	0.02	1.0	0.8	1.0
	*P*_mass_ * Pheno.type	2	73	0.02	0.3	0.8	1.0

Models were constructed as y ~ x * Pheno.type + (1|Species), where y = detected isoprene *E*_c,M_ or magnitude of isoprene *E*_c,M_ (dependent variable), x = functional trait (independent variable), and Pheno.type = leaf phenological type (interaction term). df1, degrees of freedom of interaction term; df2, degrees of freedom associated with the residual variance; F ratio, ratio of variance explained by a factor to the residual variance; *p* trait, *p*-value of x; *p* pheno, *p*-value of Pheno.type; *p* interaction, *p*-value of the interaction between x and Pheno.type. Models of detected isoprene *E*_c,M_ were performed with all trees (*n* = 154), and models of the magnitude of isoprene *E*_c,M_ were performed with trees that showed detected isoprene *E*_c,M_ (*n* = 81); both had species as a random factor. Statistically significant variables are in bold.

**Figure 3:**
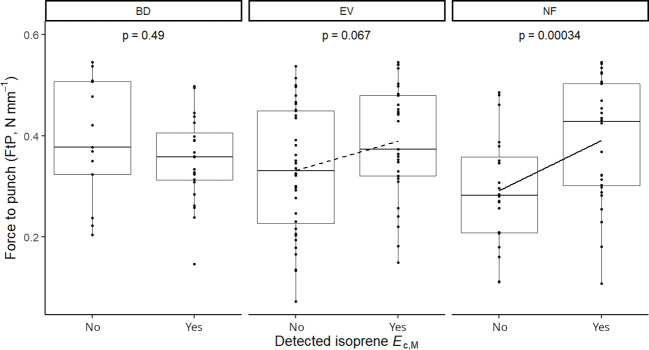
Mixed effects linear regression model of detected isoprene *E*_c,M_ (No, not detected; Yes, detected) varying as a function of force to punch (FtP, N mm^-1^) per leaf phenological type. BD, brevideciduous, trees that lost all their foliage/part of their foliage and flushed new leaves concentrated in the drier months of the year; EV, evergreen, trees that showed detectable flushing events and massively flushed new leaves, predominantly in the drier months of the year; NF, no flushing detected, evergreen trees that possibly added and lost leaves throughout the year and did not show detectable flushing crown events during the monitoring period. The model was performed with all trees as sample units (*n* = 154) and had species as a random factor. Dashed and solid lines represent p < 0.1 and p < 0.05, respectively.

**Figure 4:**
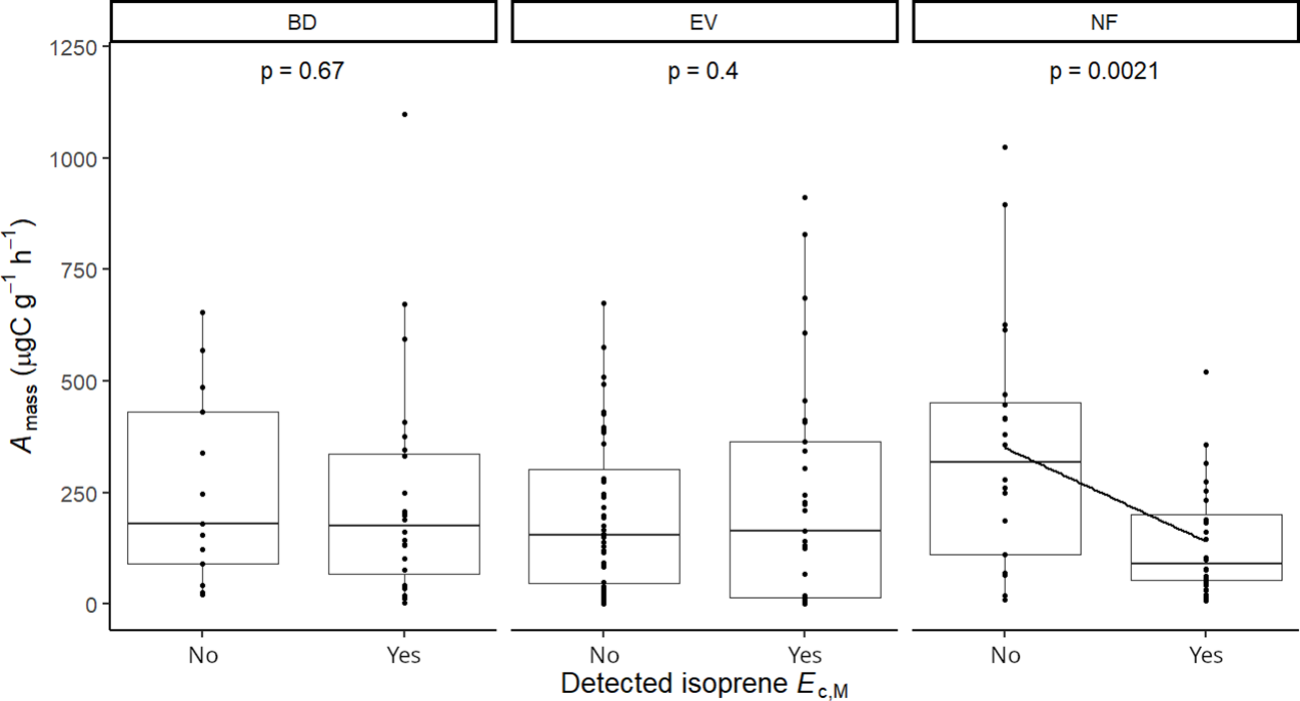
Mixed effects linear regression model of detected isoprene *E*_c,M_ (No, not detected; Yes, detected) varying as a function of photosynthesis per leaf dry mass (*A*_mass_, µg C g^-1^ h^-1^) per leaf phenological type. BD, brevideciduous, trees that lost all their foliage/part of their foliage and flushed new leaves concentrated in the drier months of the year; EV, evergreen, trees that showed detectable flushing events and massively flushed new leaves, predominantly in the drier months of the year; NF, no flushing detected, evergreen trees that possibly added and lost leaves throughout the year and did not show detectable flushing crown events during the monitoring period. The model was performed with all trees as sample units (*n* = 154) and had species as a random factor. The solid line represents p < 0.05.

**Figure 5:**
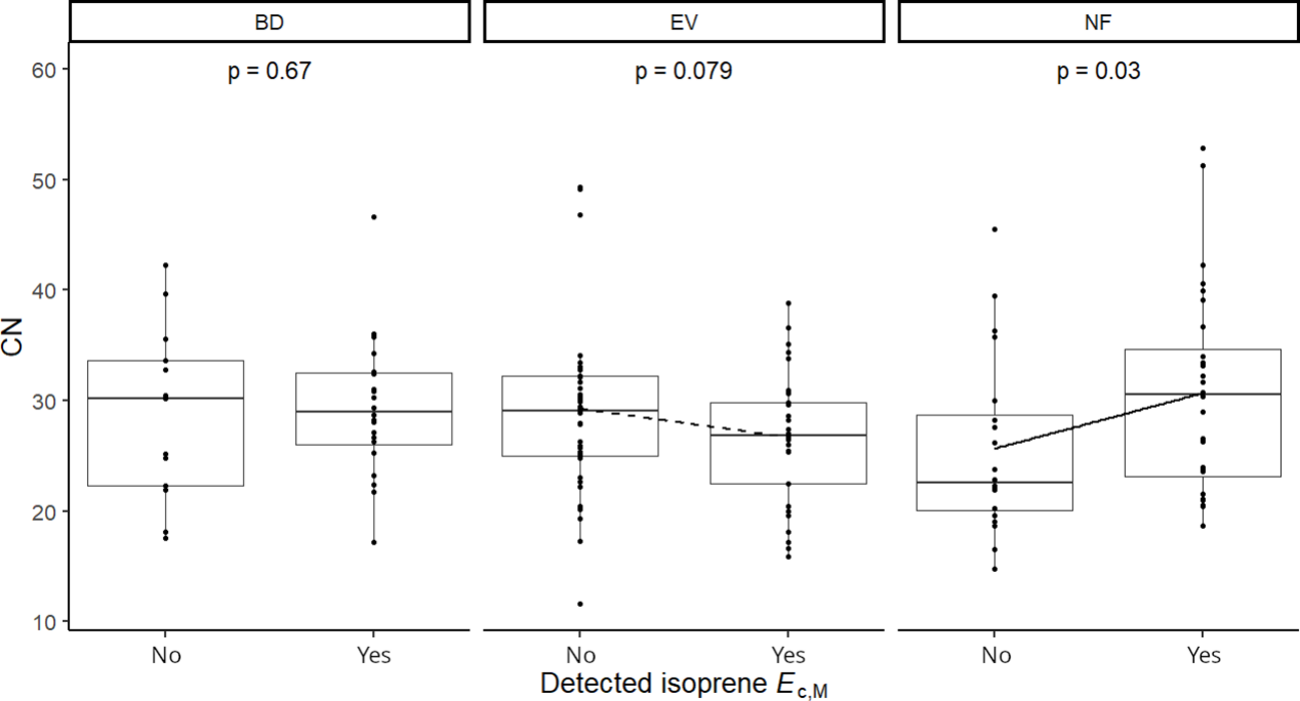
Mixed effects linear regression model of detected isoprene *E*_c,M_ (No, not detected; Yes, detected) varying as a function of carbon-to-nitrogen ratio (CN) per leaf phenological type. BD, brevideciduous, trees that lost all their foliage/part of their foliage and flushed new leaves concentrated in the drier months of the year; EV, evergreen, trees that showed detectable flushing events and massively flushed new leaves, predominantly in the drier months of the year; NF, no flushing detected, evergreen trees that possibly added and lost leaves throughout the year and did not show detectable flushing crown events during the monitoring period. The model was performed with all trees as sample units (*n* = 154) and had species as a random factor. Dashed and solid lines represent p < 0.1 and p < 0.05, respectively.

**Figure 6:**
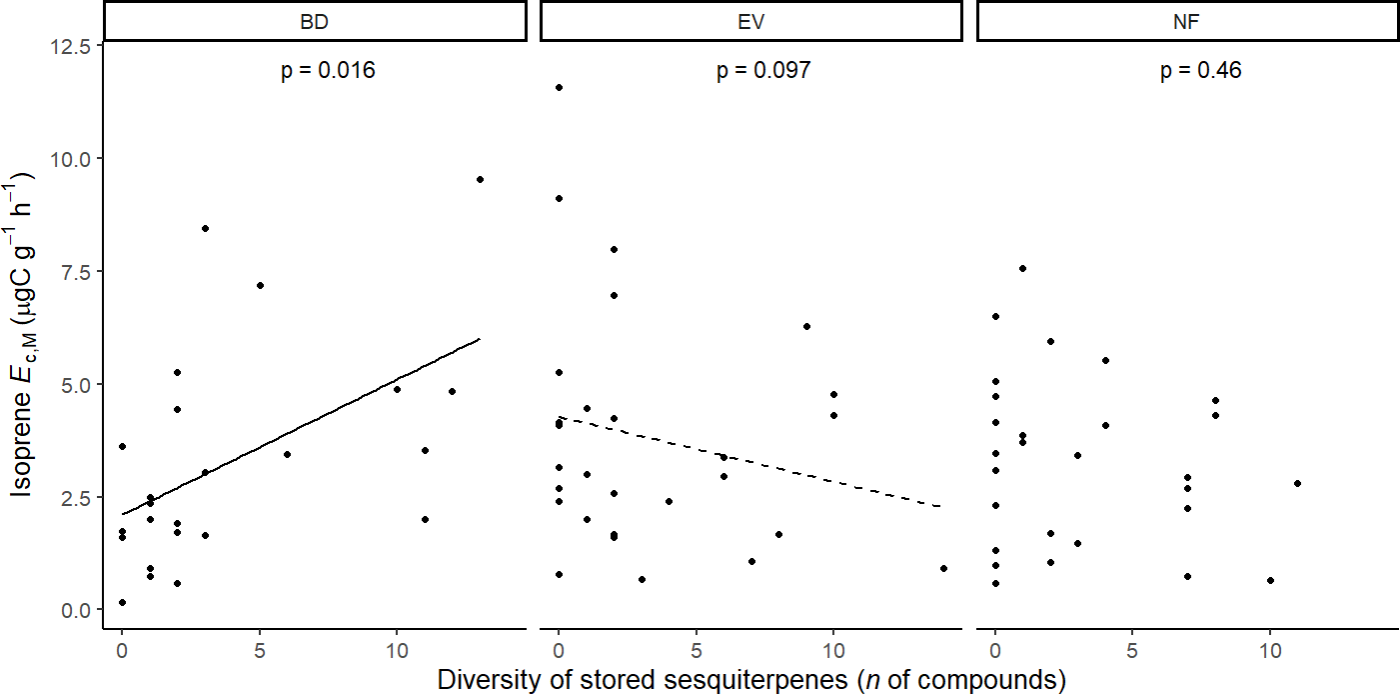
Mixed effects linear regression model of isoprene *E*_c,M_ (µg C g^-1^ h^-1^) varying as a function stored sesquiterpene diversity (*n* of compounds) per leaf phenological type. BD, brevideciduous, trees that lost all their foliage/part of their foliage and flushed new leaves concentrated in the drier months of the year; EV, evergreen, trees that showed detectable flushing events and massively flushed new leaves, predominantly in the drier months of the year; NF, no flushing detected, evergreen trees that possibly added and lost leaves throughout the year and did not show detectable flushing crown events during the monitoring period. The model was performed with all trees that showed detected isoprene *E*_c,M_ as sample units (*n* = 81), and had species as a random factor. Dashed and solid lines represent p < 0.1 and p < 0.05, respectively.

**Figure 7:**
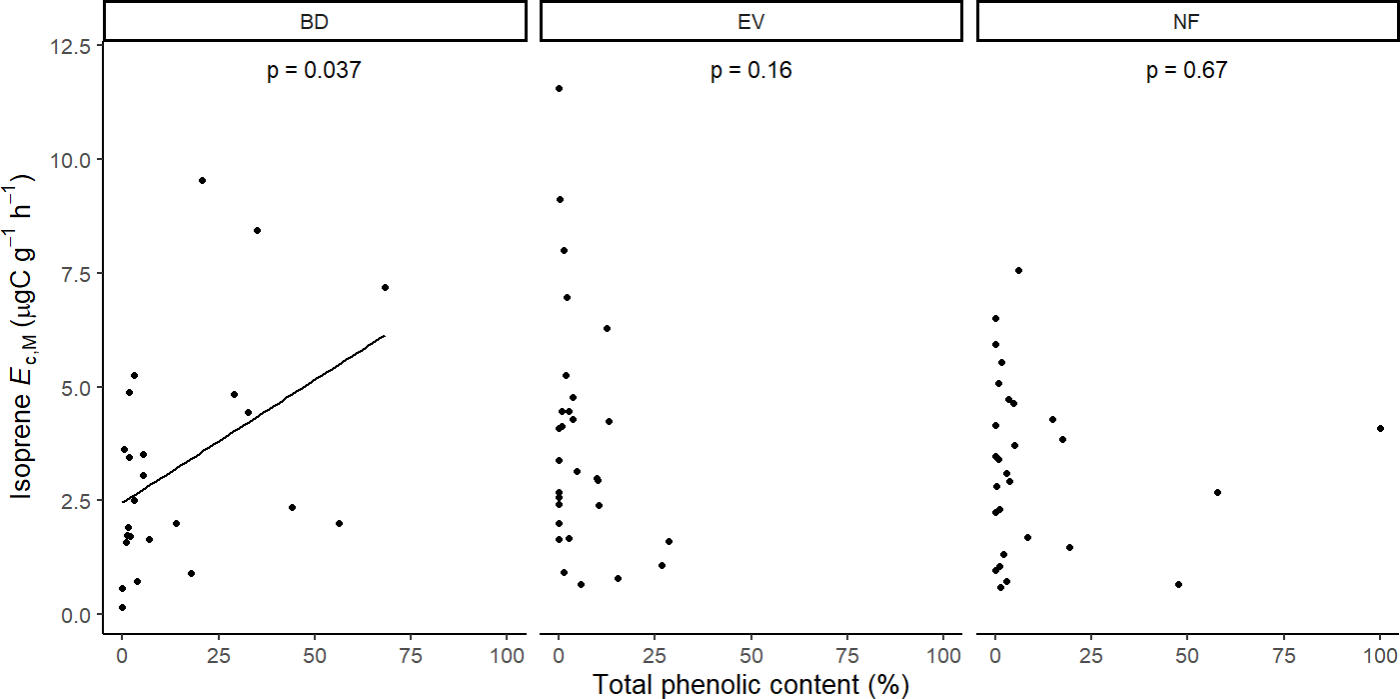
Mixed effects linear regression model of isoprene *E*_c,M_ (µg C g^-1^ h^-1^) varying as a function of total phenolics content (% of relative abundance) per leaf phenological type. BD, brevideciduous, trees that lost all their foliage/part of their foliage and flushed new leaves concentrated in the drier months of the year; EV, evergreen, trees that showed detectable flushing events and massively flushed new leaves, predominantly in the drier months of the year; NF, no flushing detected, evergreen trees that possibly added and lost leaves throughout the year and did not show detectable flushing crown events during the monitoring period. The model was performed with all trees that showed detected isoprene *E*_c,M_ as sample units (*n* = 81), and had species as a random factor. The solid line represents p < 0.05.

**Figure 8:**
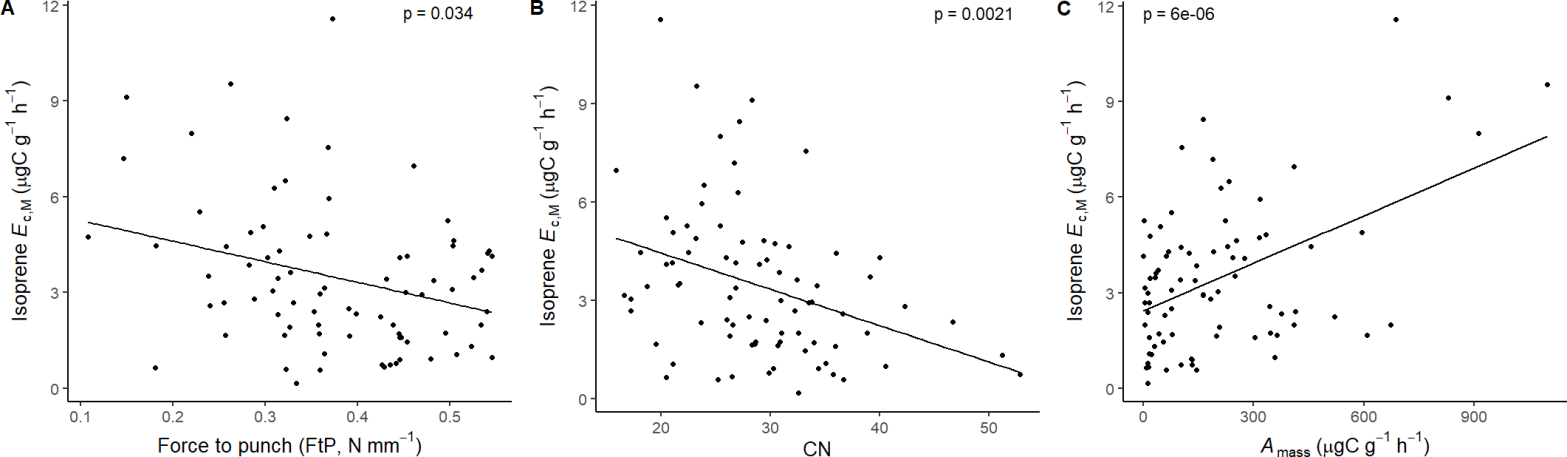
Mixed effects linear regression models of isoprene *E*_c,M_ (µg C g^-1^ h^-1^) varying as a function of **(A)** force to punch (FtP, N mm^-1^), **(B)** carbon-to-nitrogen ratio (CN), and **(C)** photosynthesis per leaf dry mass (*A*_mass_, µg C g^-1^ h^-1^). The model was performed with all trees that showed detected isoprene *E*_c,M_ as sample units (*n* = 81), and had species as a random factor. The solid lines represent p < 0.05.

Isoprene-emitting trees from the no-detectable flushing (NF) group were significantly less likely to store monoterpenes (**Table 4**). These trees also showed significantly tougher (**Figure 3**) and less photosynthetically active (**Figure 4**) leaves with a higher carbon-to-nitrogen ratio (**Figure 5**). On the other hand, isoprene emission rates in brevideciduous (BD) trees were significantly higher with higher diversity of stored sesquiterpenes (**Figure 6**) and total phenolics content (**Figure 7**). Lastly, independent of the leaf phenological type, isoprene emission rates were significantly lower with FtP and CN, while higher with *A*_mass_ (**Figure 8**).

**Table 4:** Contingency table and chi-squared (χ^2^) p-values of comparisons of proportions of detected isoprene *E*_c,M_ and detected monoterpene storage in each leaf phenological type (*n* = 154).

		No detected monoterpene storage	Detected monoterpene storage	χ^2^ p-value
EV	No detected isoprene *E*_c,M_	6	7	0.9
	Detected isoprene *E*_c,M_	13	11	
BD	No detected isoprene *E*_c,M_	24	16	0.3
	Detected isoprene *E*_c,M_	13	16	
NF	No detected isoprene *E*_c,M_	8	12	0.04
	Detected isoprene *E*_c,M_	20	8	

BD, brevideciduous, trees that lost all their foliage/part of their foliage and flushed new leaves concentrated in the drier months of the year; EV, evergreen, trees that showed detectable flushing events and massively flushed new leaves, predominantly in the drier months of the year; NF, no flushing detected, evergreen trees that possibly added and lost leaves throughout the year and did not show detectable flushing crown events during the monitoring period.

## 4 Discussion

Our study presents a unique dataset of isoprene emission capacity (*E*_c_) measurements, combined with leaf phenological type and functional - physiological, morphological, and chemical - trait data for 175 trees from 124 species of angiosperms in an upland *terra firme* Amazon Forest. Many of these measurements represent the first-ever recorded data for numerous species. Evergreen trees that flushed leaves in the drier months of the year (EV) contained more non-emitters than emitters of isoprene, and sesquiterpene storage was detected in many more trees than expected by chance. Contrary to our hypothesis, isoprene emission rates and relative abundances of stored monoterpenes and sesquiterpenes did not vary between leaf phenological types. Yet, interactions between leaf phenological type and functional traits were significantly related to the presence of isoprene emissions and variations in isoprene emission rates. These relationships revealed that, for trees that continuously produced/lost leaves (NF), isoprene emitters were less likely to store monoterpenes and had significantly tougher and less photosynthetically active leaves, with a higher carbon-to-nitrogen ratio compared to non-emitters. Meanwhile, in brevideciduous (BD) trees, isoprene emission rates were significantly higher with a higher diversity of stored sesquiterpenes and total phenolics content. Finally, independent of leaf phenological type, isoprene emission rates were higher in softer (lower FtP) leaves, with lower carbon-to-nitrogen ratios, and higher photosynthesis per dry mass. In the following sections we discuss the i) distribution of isoprene emissions and terpene storage in the different leaf phenological types, ii) the relationships between leaf phenological type, functional traits and variations in the presence of isoprene emissions and isoprene emission rates, and iii) present a summary of the results and implications for emission modeling.

### 4.1 Distribution of isoprene emission capacity and terpene storage in different leaf phenological types

We have detected isoprene emissions in 88 trees from 69 species, which corresponds to c. 50% of all trees and 55.6% of all species. Most studies so far have reported only between 20-38% of tropical tree species as isoprene emitters (Harley et al., 2004; Loreto and Fineschi, 2015), but recent studies have shown that this number can be even larger, up to 76% (Jardine et al., 2020; Mu et al., 2022). Results of the distribution of isoprene emitters between leaf phenological types showed slight differences compared to what has been observed by Gomes Alves et al. (2023) through emission probability modeling, suggesting a prevalence of non-emitters of isoprene within the group of massively flushing evergreen (EV) trees. Because evergreen trees are more dominant in the central Amazon forest compared to brevideciduous trees (Condit et al., 2000; Aleixo et al., 2019), a higher fraction of non-emitters in this group may imply lower regional fluxes in this region. However, contrary to our hypothesis, we did not see significant differences in isoprene emission rates comparing leaf phenological types. The fact that brevideciduous trees in this forest may not lose all their foliage and only remain leafless/partially leafless for shorter periods, combined with the fact that this forest is mostly composed of dry-season flushing evergreen trees (Condit et al., 2000; Aleixo et al., 2019; Mesquita Pinho, 2021), could indicate that the observed seasonality of higher isoprene emissions at the end of the dry season/early wet season is probably being driven by a higher fraction of mature leaves in the canopy, regardless of leaf phenological type (Alves et al., 2018; Gomes Alves et al., 2023).

Meanwhile, we have detected sesquiterpene storage in a significantly large number of trees, with no differences in relative abundances of monoterpene and sesquiterpene storage comparing leaf phenological types, contradicting our initial hypothesis. These results show that terpene storage is widespread regardless of leaf phenological types and emphasize how tropical tree species are more complex and do not hold to general assumptions or plant trait coordinations found in temperate tree species (Dani et al., 2014). Sesquiterpenes require more carbon for production (15C) compared to isoprene (5C), with a yield rate of secondary organic aerosol (SOA) formation that can reach up to 70% (Griffin et al., 1999b, 1999a), while for isoprene it has been reported as <6% (Kroll et al., 2005; Xu et al., 2014). Studies have observed significant temperature-induced emissions of these heavier terpenes at temperatures above 35°C (Nagalingam et al., 2023; Robin et al., in preparation). Considering that tropical forest canopies can frequently experience such high temperatures (Jardine et al., 2017; Manzi et al., 2024), the large number of sesquiterpene-storing species we observed may indicate a strong potential for higher fluxes of these compounds than previously estimated by emission models (e.g., Guenther et al., 2012), and may incur higher carbon losses to the atmosphere if the forest is under more frequent stress (e.g.; heatwaves, insect outbreaks), but more research is needed to test this.

### 4.2 Interactions between leaf phenological type, functional traits, and isoprene emission capacity

Even though isoprene emission rates did not vary between leaf phenological types, interactions between functional traits and leaf phenological types were significantly related to variations in the presence and magnitude of isoprene emissions. Much had been discussed on the roles of isoprene in increased thermotolerance (Singsaas et al., 1997; Pollastri et al., 2014, 2019) and oxidative stress protection (Vickers et al., 2009; Morfopoulos et al., 2013, 2014; Rodrigues et al., 2020), and recent research has demonstrated that the presence of isoprene emissions is related to multiple up and down regulations of gene expression, transcription factors, and protein abundance (Behnke et al., 2010; Harvey and Sharkey, 2016; Lantz et al., 2019; Zuo et al., 2019; Frank et al., 2021; Monson et al., 2021; Dani et al., 2022; Weraduwage et al., 2023; Srikanth et al., 2024). The current view is that isoprene occupies a unique metabolic position, mediating processes that govern the supply of photosynthetic substrates and the requirements for secondary metabolite products, enabling plants to allocate resources to defense while minimizing the impact on growth (Monson et al., 2021).

Isoprene-emitting trees from the NF group were less likely to store monoterpenes and had tougher and less photosynthetically active leaves (i.e., higher mechanical defense). Meanwhile, in brevideciduous trees, isoprene emission rates were higher with a higher diversity of stored sesquiterpenes and higher total phenolics content (i.e., higher chemical defense). There are only a few multi-omic studies evaluating how the presence of isoprene emissions regulates chemical and mechanical defenses (Harvey and Sharkey, 2016; Zuo et al., 2019; Monson et al., 2020, 2021). In isoprene-emitting (IE) poplar leaves, for example, the presence of isoprene emissions was associated with increased expression of genes involved in the accumulation of lignin, supporting our observation of isoprene emitters from the NF group with higher FtP and CN ratios (Monson et al., 2020, 2021). On the other hand, opposite relationships between isoprene emission and terpene accumulation have been observed: in IE poplar and tobacco leaves, the presence of isoprene emissions was related to reductions in the expression of genes and proteins involved in terpene biosynthesis (Zuo et al., 2019; Monson et al., 2020, 2021); however, Harvey and Sharkey (2016) observed increases in transcript abundances of terpene synthesis-related genes under fumigation with isoprene for *Arabidopsis* plants. As for phenolics, the observed relationship between higher isoprene emission rates and higher phenolics content in BD trees corroborates with Behnke et al. (2010) and Monson et al. (2020), which showed that the presence of isoprene emissions was associated with an upregulation in the expression of genes in the phenylpropanoid pathway with consequent increases in production of phenolic compounds.

Trees from EV and BD groups presented regular flushing events in the early and mid-dry seasons, respectively, which means that their canopies have a more homogeneous leaf age composition (Lopes et al., 2016; Gonçalves et al., 2020). In contrast, since NF trees did not show detectable flushing events and probably flushed/lost new leaves continuously throughout the years, canopies from these trees likely have greater leaf age heterogeneity. Although we did not measure visually old leaves, and this group did not show significantly lower *A*_mass_ or LMA compared to the other groups (**Supplementary Figure S2**), it is possible that we measured slightly older leaves for these trees, and isoprene emissions decrease together with photosynthesis as leaves get older (Schnitzler et al., 1997; Gomes Alves et al., 2023). On the other hand, part of the NF group could be composed of trees that did not have detectable flushing events because they flush dark green leaves that are not detected as young by the PhenoCam, hence more research at the branch or leaf levels on these trees is needed to understand the mechanisms driving their leaf renewal.

One hypothesis to explain the evolutionary drivers of leaf-out phenology in upland central Amazon forests suggests that trees flush new leaves in the dry season as a way to avoid increased herbivory pressure in the wet season, since young leaves have fewer structural defenses (i.e., softer, thinner), and are more palatable to herbivores, which are more abundant in the rainy season (Wright and van Schaik, 1994; Coley and Barone, 1996; Lopes et al., 2016). Perhaps, in brevideciduous trees, the presence of isoprene emissions could be influencing metabolic regulation towards a more functionally diverse chemical-based defense, that protects the large fractions of synchronized newly flushed and vulnerable young leaves (Coley and Barone, 1996; Lopes et al., 2016). Meanwhile, in evergreen trees that continuously flushed/lost leaves (NF), isoprene may be associated with an upregulation of defense towards a more long-term, lignin-based structural defense that supports longer leaf longevities (Monson et al., 2021). This suggests that isoprene emission in this forest could be involved in the co-regulation of a chemical-mechanical defense trade-off (van der Meijden et al., 1988; Pedersen et al., 1995) between brevideciduous (BD) and evergreen trees with continuous flushing (NF), which is reinforced by the observation that isoprene emitters in NF are also less likely to store monoterpenes.

Recent studies have demonstrated how isoprene is intrinsically interconnected with broad patterns of gene expression, and that leaf phenological types are under strong genetic control, being less of an observable trait and more of a dynamic response that results from gene-environment interactions (Satake et al., 2024). For example, studies on the seasonal expression of BVOC synthesis-related genes in two tree species of Fagaceae (*Quercus glauca* and *Lithocarpus edulis*) showed that genes downstream of the MVA pathway, involved in sesquiterpene production, had increased expression during the period that matches leaf flushing for these trees (Satake et al., 2023, 2024). Even though we did not measure visually young leaves, brevideciduous trees possibly produce new leaves earlier than evergreen trees, so it is possible that their canopies were overall composed of slightly younger leaves in comparison to other leaf phenological types, and thus had increased expression of sesquiterpene synthase genes. Considering this underlying molecular component of plasticity in leaf phenological types and associations with pathways of isoprene and sesquiterpene synthesis, perhaps our results suggest that, in this resource-abundant, species-rich, ecologically-complex upland *terra-firme* central Amazon forest, the direction of isoprene’s regulation over growth and defense is possibly being influenced by leaf phenological type, although more research is needed to test this hypothesis.

Lastly, isoprene emission rates were significantly higher with characteristic resource-acquisition traits (Wright et al., 2004), like lower mechanical resistance (low FtP), and higher nitrogen content (lower CN) and *A*_mass_. It is reasonable that, independent of leaf phenological type, emission rates from isoprene emitters would be higher with such traits that enable faster leaf metabolism, hence providing sufficient carbon uptake to support stronger emission rates (Loreto and Sharkey, 1990; Delwiche and Sharkey, 1993; Loreto et al., 1996; Magel et al., 2006; Sharkey and Monson, 2017). Still, isoprene emitters from evergreen trees were generally constrained towards more resource-conservative strategies (higher FtP and lower *A*_mass_, and CN) which, given the predominance of evergreen trees in central Amazon forests (Condit et al., 2000; Aleixo et al., 2019), emphasizes the importance of incorporating leaf phenological type when estimating regional and global fluxes.

### 4.3 Summary and implications for emission modeling

Although our results showed that isoprene emissions and terpene storage did not significantly vary between evergreen and brevideciduous trees, they revealed that interactions between traits and leaf phenological types drive variations in the presence of isoprene emissions and isoprene emission rates. Isoprene-emitting trees with no detectable flushing were less likely to store monoterpenes and had tougher and less photosynthetically active leaves, while brevideciduous trees showed higher isoprene emission rates with a higher diversity of stored sesquiterpenes and total phenolics content. Recent studies have revealed that isoprene is an integrative compound that co-regulates both growth and defense responses by promoting changes in gene expression patterns and protein abundances. Our results perhaps suggest that the direction of this co-regulation is influenced by leaf phenological types, and that isoprene emissions participate in co-regulating a chemical-mechanical defense trade-off between brevideciduous and evergreen trees with continuous flushing in central Amazonia. Moreover, we detected isoprene emissions and sesquiterpene storage in a greater number of trees than expected, which indicates a greater potential for emissions of these compounds than previously thought (Harley et al., 2004; Guenther et al., 2012; Loreto and Fineschi, 2015).

Isoprene and sesquiterpene emissions, directly and indirectly, influence atmospheric processes and cloud formation, with sesquiterpenes having a yield rate of particle formation almost 10 times that of isoprene (Griffin et al., 1999b, 1999a; Kroll et al., 2005; Xu et al., 2014). Warmer climates might favor the predominance of thermotolerant isoprene-emitting trees (Singsaas et al., 1997; Pollastri et al., 2014, 2019; Taylor et al., 2018), and increased heat stress and herbivore outbreaks can induce stronger sesquiterpene emissions (Nagalingam et al., 2023; Robin et al., in preparation), but the exact effects of current global climate changes and multiple stressors (e.g. extreme heat events, more frequent and intense droughts and flooding, elevated CO_2_ and O_3_) on forest-atmosphere emission feedbacks are uncertain (Yáñez-Serrano et al., 2020; Satake et al., 2024). The Amazon forest is the greatest source of volatile isoprenoid emissions to the atmosphere (Jardine et al., 2020; Mu et al., 2022), and a better understanding of the dynamics between emissions, leaf phenological types and functional traits in this forest is essential to provide a more mechanistic understanding of emissions and improve their representation in models.

## Data Availability

The datasets presented in this study can be found in online repositories. The names of the repository/repositories and accession number(s) can be found below: 10.17871/atto.363.7.1695.
