# Supplementary material for: Interactions between leaf phenological type and functional traits drive variation in isoprene emissions in central Amazon forest trees
Source: Front Plant Sci. 2024 Dec 24;15:1522606. doi: 10.3389/fpls.2024.1522606 (PMC11703902; doi:10.3389/fpls.2024.1522606)
Supplement: Supplementary file 1 [file Table1.docx]

## *Supplementary Material*

# **1. Supplementary Figures**

**Fig. S1** Heatmap of standardized residuals from chi-squared (χ²) comparisons between observed and expected proportions of detected and non-detected isoprene *E*_c,A_ in each leaf phenological type (*n* = 175) (Main text, Fig 1a). Standardized residuals indicate that there are significantly fewer isoprene detections (more negative residuals) and significantly more non-detections (more positive residuals) than expected by chance in the EV group. BD = brevideciduous, trees that lost all their foliage/part of their foliage and flushed new leaves concentrated in the drier months of the year; EV = evergreen, trees that showed detectable flushing events and massively flushed new leaves, predominantly in the drier months of the year; NF = no flushing detected, evergreen trees that possibly added and lost leaves throughout the year and did not show detectable flushing crown events during the monitoring period (Botía et al., 2022).


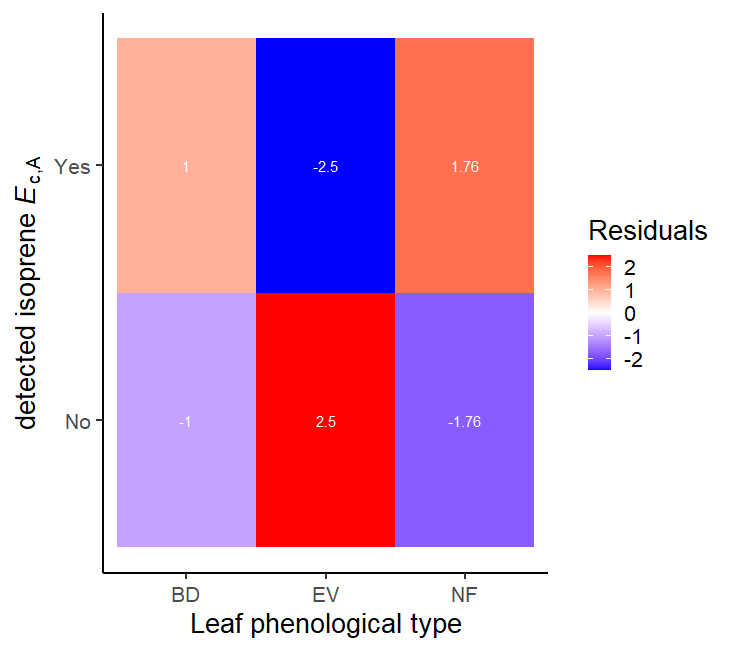


**Fig. S2** Comparison of the magnitude of (a) photosynthesis per leaf dry mass (*A*_mass_, µg C g^-1^ h^-1^) and (b) leaf dry matter content (LMA, g cm^-2^) between leaf phenological types. BD = brevideciduous, trees that lost all their foliage/part of their foliage and flushed new leaves concentrated in the drier months of the year; EV = evergreen, trees that showed detectable flushing events and massively flushed new leaves, predominantly in the drier months of the year; NF = no flushing detected, evergreen trees that possibly added and lost leaves throughout the year and did not show detectable flushing crown events during the monitoring period. Pair-wise comparisons are mixed effects models that include all trees as sample units (*n* = 154) and have species as a random effect.


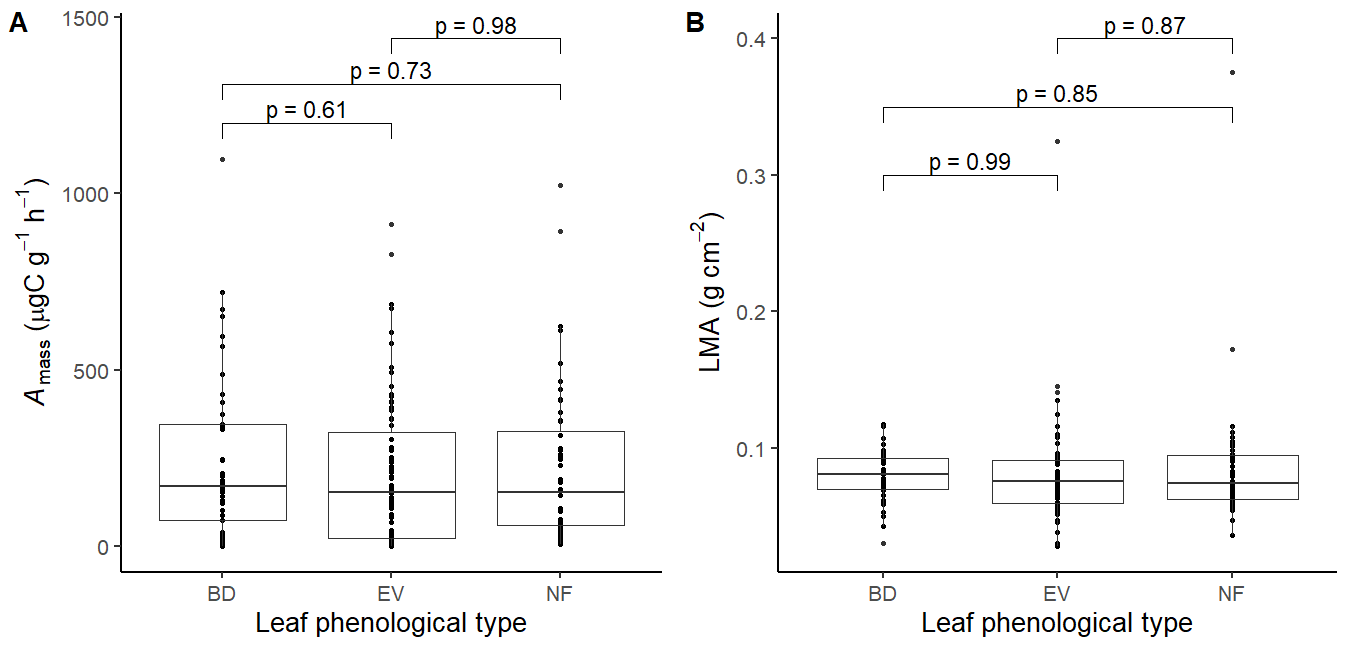


# **2. Supplementary Tables**

**Table S1** Multi-component calibration mixture in synthetic air. Uncertainty is a conservative estimate of the combination of gravimetric preparation and analysis uncertainties.

| **Compound** | **CAS#** | **Concentration (ppb)** | **Uncertainty** |
| --- | --- | --- | --- |
| Formaldehyde | 50-0-0 | 992 | 5% |
| Acetaldehyde | 75-07-0 | 426 | 5% |
| Methanol | 67-56-1 | 521 | 5% |
| Ethanol | 64-17-5 | 528 | 5% |
| Acetonitrile | 75-05-8 | 523 | 5% |
| Acetone | 67-64-1 | 497 | 5% |
| Isoprene | 78-79-5 | 468 | 5% |
| DMS | 75-18-3 | 487 | 5% |
| α-Pinene | 80-56-8 | 488 | 5% |
| Camphene | 79-92-5 | 504 | 5% |
| β-Pinene | 18172-67-3 | 512 | 5% |
| α-Phellandrene | 4221-98-1 | 432 | 5% |
| 3-Carene | 13466-78-9 | 458 | 5% |
| Limonene | 5889-54-8 | 489 | 5% |
| o-Cymene | 527-84-4 | 481 | 5% |
| γ-Terpinene | 99-85-4 | 476 | 5% |
| β-Caryophyllene | 87-44-5 | 98.3 | 5% |
| α-Humulene | 6753-98-6 | 95.2 | 5% |

**Table S2** Statistical parameters of statistically significant mixed effects linear regression models of detected isoprene emission capacity per dry mass (Ec,M) and magnitude of isoprene Ec,M. Models of detected isoprene Ec,M were performed with all trees (n = 154), and models of the magnitude of isoprene Ec,M were performed with trees that showed detected isoprene Ec,M (n = 81); both had species as a random factor. Full names and units of variables are in the main text (Table 1). Pheno.type = leaf phenological type (BD = brevideciduous, trees that lost all their foliage/part of their foliage and flushed new leaves concentrated in the drier months of the year; EV = evergreen, trees that showed detectable flushing events and massively flushed new leaves, predominantly in the drier months of the year; NF = no flushing detected, evergreen trees that possibly add and lose leaves throughout the year and did not show detectable flushing crown events during the monitoring period), SE = standard error, CI = confidence interval.

| **Dependent variable** | **Independent variable** | **Pheno.type** | **slope** | **SE** | **CI** | **p-value** |
| --- | --- | --- | --- | --- | --- | --- |
| Detected isoprene *E*_c,M_ | Presence of stored monoterpenes | BD | -0.08 | 0.17 | -0.42 – 0.26 | 0.64 |
|  |  | EV | 0.15 | 0.12 | -0.09 – 0.40 | 0.22 |
|  |  | **NF** | **-0.31** | **0.15** | **-0.60 – -0.02** | **0.03** |
|  | FtP | BD | -0.03 | 0.04 | -0.10 – 0.05 | 0.049 |
|  |  | EV | 0.05 | 0.03 | -0.004 – 0.10 | 0.067 |
|  |  | **NF** | **0.11** | **0.03** | **0.05 – 0.17** | **0.00034** |
|  | *A*_mass_ | BD | -32.0 | 75.7 | -181.5 – 117.6 | 0.67 |
|  |  | EV | 46.4 | 55.2 | -62.7 – 155.4 | 0.4 |
|  |  | **NF** | **-200.1** | **63.9** | **-326.5 – -73.7** | **0.0021** |
|  | CN | BD | 1.03 | 2.42 | -3.76 – 5.82 | 0.67 |
|  |  | EV | -2.92 | 1.65 | -6.19 – 0.35 | 0.079 |
|  |  | NF | 4.22 | 1.93 | 0.41 – 8.04 | **0.03** |
| Magnitude of isoprene *E*_c,M_ | Stored sesquiterpene diversity | **BD** | **0.25** | **0.10** | **0.05 – 0.46** | **0.016** |
|  |  | EV | -0.17 | 0.10 | -0.37 – 0.03 | 0.097 |
|  |  | NF | -0.09 | 0.12 | -0.33 – 0.15 | 0.46 |
|  | Total phenolic content | **BD** | **0.05** | **0.02** | **0.003 – 0.09** | **0.037** |
|  |  | EV | -0.07 | 0.05 | -0.17 – 0.03 | 0.16 |
|  |  | NF | -0.008 | 0.02 | -0.05 – 0.03 | 0.67 |

# **3. Supplementary Methods**

**Methods S1** Leaf morphological trait measurements.

All morphological trait measurements and calculations were performed according to published protocols (Pérez-Harguindeguy et al., 2013). We weighed the leaves to measure fresh mass and used a micrometer to measure leaf thickness (LT) and a table scanner to measure leaf area. Leaves were dried in an oven for 72 h at 60 ºC and weighed again to measure dry mass. We measured force to punch (FtP) using a Pesola® scale (Medio spring scale, item n. 40300) modified with a pressure set (accessory for Medio-scales, item n. 4.004) and quantified it in N mm^-1^ considering that 1 kg of force is equivalent to 9.81 N, then FtP (N mm^-1^) = ((FtP (g) / 1000) * 9.81) / 5.35 (mm) (Pérez-Harguindeguy et al., 2013). We analyzed images of scanned leaves with ImageJ software (Schneider et al., 2012) to obtain leaf area. We calculated specific leaf area (SLA) as the ratio of leaf area to leaf dry mass. We did not include petioles in the SLA calculation since they can be quite large for rainforest species and are usually more related to leaf positioning rather than biomass efficiency (Poorter et al., 2018). We calculated leaf dry matter content (LDMC) as the ratio of leaf dry mass to fresh mass, and leaf mass per area (LMA) as 1 / SLA.

**Methods S2** Analysis of terpene storage.

Leaves collected for terpene storage analysis were immediately frozen in liquid nitrogen and taken to the lab in Manaus. We analyzed monoterpenes and sesquiterpenes from ~2 g of fresh leaf material macerated in liquid nitrogen and transferred to a 20 mL glass vial. Samples were analyzed via gas chromatography with a headspace mass-spectrometry detector (Headspace/GC-MS) system at the Chemical Analysis Lab (State University of Amazonas - UEA). Before each injection, the automatic sampler (AOC-6000 plus, Shimadzu, Kyoto, Japan) transferred the glass vial to the headspace oven and the vial was heated to 150 °C for 20 min at 250 rpm. Following that, the gas phase of the sample was extracted with a hermetic needle at 150 ºC and injected into the column of a gas chromatography-mass spectrometer GC-MS-TQ8050 NX (Shimadzu, Kyoto, Japan). The injection was split (1:10), and the trap was heated to 200 ºC while backflushing with a carrier gas (helium) at a flow rate of 5.2 mL min-1 (linear speed of 37.4 cm s-1 and pressure of 53.5 kPa) directed into the column (SH-I-5Sil MS, 5% diphenyl / 95% dimethyl pol, 30.0 m length x 0.25 mm inner diameter x 0.25 μm film thickness). The oven ramp temperature was programmed with an initial hold of 5 min at 35 ºC, followed by an increase to 280 ºC at a rate of 5 ºC min-1 followed by a hold at 280 ºC for 36 min. The interface was at 290 °C and the ion source was at 200 °C.

A Shimadzu gas quality workstation (GC-MS solution v. 4.53) and NIST20 Mass Spectral Library were used to analyze the chromatographic data. The peak integration parameters were set as follows: slope (S) was 100, peak width at half-height (W) was 3, drift (D) was 0, parameter change time (T) was 1000, minimum peak area (M) was 1000, smoothing (F) was 1, and smoothing peak width at half-height (O) was 1. With these peak integration parameters, chromatographic peaks were automatically integrated. The mass spectrum of each peak was compared with the standard spectrums in the NIST20 spectrum library, and compounds with a similarity score of more than 90 were selected. We calculated values as percentages of relative abundance of stored monoterpenes and stored sesquiterpenes by summing the peak areas of stored monoterpenes (sum of stored monoterpenes) and stored sesquiterpenes (sum of stored sesquiterpenes) found in a given sample and normalizing each sum by the largest sum observed in the dataset for each group of compounds.

**Methods S3** Leaf stable C isotope and elemental analyses.

Determination of foliar δ13C composition was performed by the Stable Isotope Analysis Lab (BGC-IsoLab, MPI-BGC) and the values are expressed relative to Pee Dee Belemnite. Leaf carbon and nitrogen contents were determined by the Routine Measurements & Analyses Lab (RoMA, MPI-BGC) with the elemental analyzer "varioEL" (Elementar Analysensysteme GmbH, Elementar-Straße 1, D-63505 Langenselbold, Germany). Leaf phosphorus content was determined by the Spectrometry Laboratory (SpecLab, MPI-BGC) via microwave-assisted high-pressure digestion (Multiwave GO plus, Anton Paar, Graz, Austria) with the addition of 7 mL 65% Suprapur HNO3 (Merck, Darmstadt, Germany) using a rotor 12HVT50 with reaction vessels made of TFM (tetrafluor-modified polytetrafluoroethylene). Maximum microwave power was 1000 W and working pressure 20 bar. In general, full digestion was achieved in about 50 min, including cooling of the hot vessels at zero microwave power. Digests were filtered, transferred to 50 mL PE vessels, and filled to the mark with ultrapure water (Millipore, Eschborn, Germany). Digested solutions were analyzed by ICP-OES “Arcos”, (Spectro, Kleve, Germany) equipped with a 27.12 MHz free-running LDMOS generator and ORCA optical system. The analyses were carried out referring to the wavelength of 177.5 nm for phosphorus according to DIN EN ISO 11885 and the calibration was based on a single-element standard issued by Merck, Darmstadt, Germany.

**Methods S4** Analysis of total phenolics content.

Phenolic compounds were extracted from 30 mg of ground freeze-dried sample with 1 ml methanol, and the mixture was vortexed for 10 min, bead-beaten (MP Biomedicals, Santa Ana, CA, USA) and centrifuged for 10 min at 13,000 g. The supernatant was collected, and the pellet was re-extracted with 0.5 ml methanol. Both supernatants were combined and analyzed using a combined system of high-performance liquid chromatography with ultraviolet spectroscopy (HPLC-UV, Agilent 1100 Series; Agilent Technologies, Waldbronn, Germany) at the Department for Biochemistry of the MPI-CE. Extracts were analyzed by HPLC-UV using a C-18 reversed-phase column (Nucleodur Sphinx RP, 250 x 4.6 mm, 5 μm particle size; Macherey-Nagel, Düren, Germany). The mobile phase consisted of 0.2% formic acid (v/v) (solvent A) and acetonitrile (solvent B) used in gradient mode at a flow rate of 1 mL min-1 at 25 °C. The gradient was as follows: 90% A (0 min), 10-60% B (20 min), 60-100% B (0.1 min), 100% B (2.9 min), and 90% A (4.9 min). The eluent was monitored by a photodiode array detector at 280 nm and 330 nm.

Peaks of phenolic compounds in the chromatograms obtained at UV 280 and 330 nm were automatically integrated using the Agilent OpenLAB CDS LC ChemStation v. 10.0 (Agilent Technologies, Inc. 2010-2020), excluding the first and last 5 min of the run. We filtered peaks that were detected only at 280 or 330 nm, and peaks that were simultaneously detected at 280 and 330 nm. For peaks that were detected in both wavelengths, we selected the signal of the peak with the highest value. After this filtering process, we added the values of peak areas for each sample and normalized values by the maximum value found in the dataset multiplied by 100. Because our study deals with a great number of tropical tree species (124) that have not been previously analyzed for phenolic composition, we were not able to use a method that required the identification and quantification of individual phenolic compounds present in the samples. The HPLC-UV method we used allowed for an estimate of the total phenolic content found in the samples by providing a measure of the abundance of methanol-soluble compounds present with UV-absorption bands indicating phenolic ring systems (Huang et al., 2023).

**Supplementary Material References**

Botía, S., Komiya, S., Marshall, J., Koch, T., Gałkowski, M., Lavric, J., et al. (2022). The CO2 record at the Amazon Tall Tower Observatory: A new opportunity to study processes on seasonal and inter-annual scales. *Glob Chang Biol* 28, 588–611. doi: 10.1111/gcb.15905

Huang, J., Hartmann, H., Ogaya, R., Schöning, I., Reichelt, M., Gershenzon, J., et al. (2023). Hormone and carbohydrate regulation of defense secondary metabolites in a Mediterranean forest during drought. *Environ Exp Bot* 209. doi: 10.1016/j.envexpbot.2023.105298

Pérez-Harguindeguy, N., Díaz, S., Garnier, E., Lavorel, S., Poorter, H., Jaureguiberry, P., et al. (2013). New handbook for standardised measurement of plant functional traits worldwide. *Aust J Bot* 61, 167–234. doi: 10.1071/BT12225

Poorter, L., Castilho, C. V., Schietti, J., Oliveira, R. S., and Costa, F. R. C. (2018). Can traits predict individual growth performance? A test in a hyperdiverse tropical forest. *New Phytologist* 219, 109–121. doi: 10.1111/nph.15206

Schneider, C. A., Rasband, W. S., and Eliceiri, K. W. (2012). NIH Image to ImageJ: 25 years of image analysis. *Nat Methods* 9, 671–675. doi: 10.1038/nmeth.2089
